# INSIGHT FROM RECRUITING DYADS OF INDIVIDUALS WITH DEMENTIA AND CARE PARTNERS FOR A MULTI-SITE ORAL CARE INTERVENTION

**DOI:** 10.1093/geroni/igae098.4369

**Published:** 2024-12-31

**Authors:** Jing Wang, Shahrzad Siamdoust, Sophie Zhijing Xu, Ruth Anderson, Brenda Plassman, Donald Bailey, Bei Wu

**Affiliations:** The University of New Hampshire, Durham, New Hampshire, United States; New York University, New York, New York, United States; New York University, New York, New York, United States; Duke University, Durham, North Carolina, United States; Duke University Medical Center, Durham, North Carolina, United States; Duke University, Durham, North Carolina, United States; New York University, New York, New York, United States

## Abstract

Maintaining oral health is essential for individuals with dementia, yet they often need support. We sought to identify lessons learned during the recruitment process for an intervention designed to teach care partners skills to guide individuals with mild dementia in proper oral hygiene techniques and provide reminders to practice oral hygiene care. Throughout the study, research team members from two research sites met regularly to discuss their experiences in recruiting participants. Notes from these meetings served as the basis for identifying key challenges and effective strategies. Recruitment involved direct patient engagement, collaboration with dementia research centers and community organizations, and the use of electronic health record systems, including MyChart. Recruitment coordinators connected study personnel with organizations and support groups, thereby broadening the recruitment base. The most effective strategies were leveraging established relationships or building new ones with clinical dementia experts to access a clinically well-characterized patient pool and presenting at support groups within dementia-focused community organizations to build connections and disseminate information about the study. Barriers to recruitment included the reduced frequency of patient visits to clinics during the COVID-19 pandemic, which limited in-person recruitment opportunities, as well as the time required to establish new relationships with non-affiliated hospitals and community organizations. Facilitating factors included building trust-based relationships with medical and community facilities, diversifying recruitment channels, maintaining flexible and clear communication, and customizing strategies for different contexts. In conclusion, successful recruitment for this intervention required a multifaceted approach that combined strong partnerships with flexibility and tailored communication strategies.

